# Performance enhancement of classifiers through Bio inspired feature selection methods for early detection of lung cancer from microarray genes

**DOI:** 10.1016/j.heliyon.2024.e36419

**Published:** 2024-08-17

**Authors:** Karthika M S, Harikumar Rajaguru, Ajin R. Nair

**Affiliations:** a Department of Information Technology, Bannari Amman Institute of Technology, Sathyamangalam, India; b Department of Electronics and Communication Engineering, Bannari Amman Institute of Technology, Sathyamangalam, India

**Keywords:** Lung cancer, Microarray genes, Adenocarcinoma, Mesothelioma, Hilbert transform, Detrend fluctuation analysis, Least square linear regression, Elephant herd optimization, Cuckoo search

## Abstract

Gene expression in the microarray is assimilated with redundant and high-dimensional information. Moreover, the information in the microarray genes mostly correlates with background noise. This paper uses dimensionality reduction and feature selection methods to employ a classification methodology for high-dimensional lung cancer microarray data. The approach is enforced in two phases; initially, the genes are dimensionally reduced through Hilbert Transform, Detrend Fluctuation Analysis and Least Square Linear Regression methods. The dimensionally reduced data is further optimized in the next phase using Elephant Herd optimization (EHO) and Cuckoo Search Feature selection methods. The classifiers used here are Bayesian Linear Discriminant, Naive Bayes, Random Forest, Decision Tree, SVM (Linear), SVM (Polynomial), and SVM (RBF). The classifier's performances are analysed with and without feature selection methods. The SVM (Linear) classifier with the DFA Dimensionality Reduction method and EHO feature selection achieved the highest accuracy of 92.26 % compared to other classifiers.

## 1 Introduction

Early detection of lung cancer can lead to more effective treatment and a higher chance of survival. According to WHO (World Health Organization), lung cancer is one of the leading causes of death, as indicated by Chang et al. [1]. So, prematurely detecting cancer symptoms is important for reducing the mortality rate. More treatment options are often available when the cancer is found early, and they are more likely to improve the survival rate. Early detection of lung cancer can also help prevent cancer from spreading metastasis to other body parts, improving a patient's prognosis, elaborated by Blandin et al. [2]. The clinical and biopsy procedure of cancer performed for cancer detection is a common method to identify the presence of cancer. The following are some significant disadvantages that disfavour this traditional procedure, as provided in Overman et al. [3]. Both procedures can cause pain and discomfort, especially during the biopsy procedure when a tissue sample is taken from the body. There is a risk of infection during both procedures, especially if the biopsied area is not properly sanitised. Biopsy procedures can result in bleeding at the biopsy site, which may require additional treatment. Also, these procedures can be expensive and may not be covered by insurance, leading to financial burdens for patients, as described in Miller et al. [4]. The Microarray Gene Expression Data (MGED) analysis solves the problems faced by conventional diagnosis methods, as explored in Pollack et al. [5]. Microarray technology allows for the simultaneous analysis of thousands of genes, providing a comprehensive view of gene expression patterns in a single experiment. Microarray analysis is non-invasive, meaning no tissue samples are taken from the body, reducing the risks associated with biopsy procedures. Microarray analysis allows for early detection of changes in gene expression patterns, indicating the presence of cancer before clinical symptoms are evident, as mentioned in Bhawal et al. [6]. Microarray technology provides highly sensitive and accurate gene expression measurements, reducing the risk of false negatives and positives compared to traditional clinical and biopsy procedures. Microarray analysis requires very small amounts of tissue, making it an efficient use of sample material. In microarray technology, the genetic makeup of lung cancer cells is analysed. A microarray is a small glass slide or chip coated with thousands of tiny spots of DNA. Each spot contains a specific gene or set of genes. This method allows it to simultaneously analyse the expression levels of thousands of genes in a single sample, as sermonised in Xiang et al. [7]. The data generated by MGE is used to identify genes that are differentially expressed between two or more samples, such as normal and cancerous tissue, or to identify genetic variations, such as single nucleotide polymorphisms (SNPs). The MGE detection is widely used in genomics, transcriptomics, and clinical research.

### 1.1 Review of related works

As stated by Tarca et al. [8], there are two main approaches to analysing microarray data: clinical and semantic. Clinical methods focus on the statistical analysis of gene expression data to identify differentially expressed genes and gene pathways associated with a particular disease or condition. These methods typically involve using linear models, clustering algorithms, and pathway analysis tools to identify up or down regulated genes in a specific biological condition. On the other hand, semantic methods use ontologies and controlled vocabularies to annotate and interpret microarray data. These methods describe gene functions, molecular interactions, and cellular processes using standard terminology. Semantic methods can identify functional relationships between differentially expressed genes and generate hypotheses about the underlying biological mechanisms that drive gene expression changes in a particular biological system, as described in Taylor et al. [9]. Clinical methods are best suited for identifying specific genes and pathways associated with a particular biological condition. In contrast, semantic methods are more useful for generating hypotheses about the underlying biological mechanisms that drive gene expression changes in a complex biological system, as indicated in Hristovski et al. [10].

**Fig. 1:**
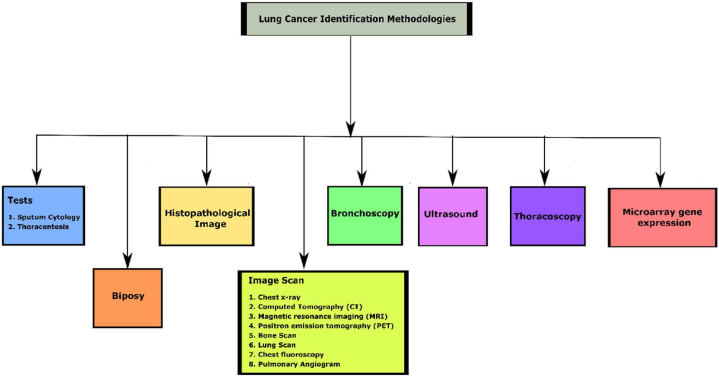
Tree diagram representation for various lung cancer methodologies.

For more than a century, different methodologies have been adopted in detecting lung cancer, varying from clinical procedures, radiation methodology and imaging methods. The thirst to identify better investigation procedures leads to early detection of lung cancer, which is evident from the CT scan technology. Sputum Cytology is a method used to analyse the sputum to check the existence of cancer cells. Bronchoscopy uses lung tissue samples to examine the airways by tiny, flexible tube and camera setup. Histopathological image-based microscopic analysis of the cancer tissues identifies malignant and benign cells. The biopsy is another invasive method to collect suspicious body samples to detect cancer cells. An ultrasound test uses fine elastic tubes to look within the breathing tube's airways with an ultrasound probe to visualize the internal organs. Like ultrasound, Thoracoscopy is a procedure to visualize the internal organs with a light and small video camera for assisting thoracic surgery. Further, the emergence of computer-assisted diagnostic methods like MRI, PET, bone scan, lung scan, chest fluoroscopy and pulmonary angiogram provides better investigation on lung cancer detection. A tree diagram representing various lung cancer identification methodologies is shown in Fig. 1.

The limitation of clinical methods is that they rely on statistical models that may be oversimplified and cannot capture the underlying biological mechanisms. Hence it may be inappropriate for the complex nature of gene expression data. Likewise, semantic methods rely on ontologies and controlled vocabularies, which may only sometimes fully capture the complexity and diversity of gene expression data. Also, semantic methods may be limited by the availability and quality of existing ontologies and the ability to annotate genes with relevant functional information accurately. To overcome the limitations of clinical and semantic methods, new approaches like machine learning has emerged in recent years, as specified by Khalsan et al. [11]. These approaches can be utilised to identify complex relationships and patterns in MGED that may be difficult to identify using traditional statistical methods. Linear programming (LP) and nonlinear programming (NLP) method-based optimization techniques are suggested by Antonov et al. [12] and Thomas et al. [13], respectively, for gene-based analysis. In gene-based analysis, the goal is to identify genetic variants associated with a particular trait or disease outcome. Through LP and NLP, the statistical models are optimized, and associations are identified. Also, many bioinformatics-based methods for gene-based analysis use computational tools to process and analyse large amounts of genomic data to identify genetic variants associated with a particular trait or disease outcome.

Genome-Wide Association Studies (GWAS), as mentioned by Visscher et al. [14], are a widely used method for identifying genetic variants associated with a particular trait or disease outcome. This method involves comparing the genomes of large groups of individuals with and without the trait of interest to identify genetic differences that are more common in individuals with the trait. Transcriptome analysis, as elaborated in Trapnell et al. [15], involves analyzing the expression patterns of all genes in a cell or tissue. This method can be used to identify genes that are differentially expressed between individuals with and without a particular trait or disease outcome. As Alipanahi et al. [16] discussed, machine learning is a computational approach that involves training models on large amounts of genomic data to identify patterns or predict outcomes. Machine learning can be used for gene-based analysis to identify genetic variants associated with a particular trait or disease outcome. Subramanian et al. [17] discuss the pathway analysis involving the interactions between genes and proteins in a particular biological pathway or process. This method can be used to identify pathways that are deregulated in individuals with a particular trait or disease outcome.

There are many traditional algorithms in literature like Fisher's Linear Discriminant Analysis (LDA), Linear regression, Non-linear regression, Gaussian Mixture Models (GMM), Logistic regression and AdaBoost. Chiaretti et al. [18] classified lymphocytic leukemia samples using LDA based on gene expression profiles. They were found to be effective in identifying genes that were significantly differentially expressed between the classes. But, LDA assumes Gaussian distribution, which may not be true for gene expression data. MGED often has thousands of genes, resulting in high dimensionality and complicating LDA computation. Also, LDA is sensitive to outliers and can be impacted by noisy or irrelevant data. Jin et al. [19] used linear regression for analysis of gene expression. But, Linear regression assumes that the relationship between the dependent variable and independent variables is linear, which may not be the case for gene expression data. Linear regression is prone to overfitting, especially when the number of independent variables is high relative to the sample size. Linear regression assumes that the independent variables are not highly correlated with each other, which is not always the case for gene expression data. Xuan et al. [20] perform the normalisation of MGED by non-linear regression through an iterative method. But, non-linear regression requires selecting an appropriate non-linear function, which can be challenging when working with complex gene expression data. Non-linear regression can get stuck in local minima, resulting in suboptimal models. Non-linear regression can be sensitive to outliers, significantly impacting the results. Non-linear regression is prone to overfitting, especially when the number of parameters in the model is high relative to the sample size. Wang et al. [21] integrated GMM with Information-Gain (IG) for gene selection. But GMM assumes data to be Gaussian, which is incorrect with the case for MGED. GMM is sensitive to outliers and can be impacted by noisy or irrelevant data. It also requires very large sample sizes to estimate the covariance matrix accurately. Likewise, Logistic regression and AdaBoost also have limitations with gene expression data due to the assumption of data independence, overfitting problem and model interpretation.

As sermonised in Libbrecht et al. [22], the MGED is integrated with Machine learning methods can unveil patterns and associations in the data which is cumbersome through traditional statistical methods. One common approach is to use supervised machine learning algorithms, such as decision trees, random forests, or support vector machines, to classify samples based on their MGE profiles. The profiles can identify genes that predict a particular phenotype, such as cancer subtype or treatment response. Unsupervised machine learning algorithms, such as clustering and Dimensionality Reduction (DR), are used to identify patterns and subgroups in MGED without prior information of the sample class labels. Additionally, Feature Selection (FSEL) algorithms can bring out the subset of genes and provide significant information, as mentioned in Xing et al. [23]. FSEL can be useful for reducing the dimensionality of the data and improving the performance of machine learning models. So the machine learning techniques used on MGED are an active area of research, and new methods and techniques are continuously being developed.

Adenocarcinoma (Adeno) and Mesothelioma (Meso) are both types of lung cancer that can be dangerous if not detected and treated early. The differentiation between these two types of lung cancer is elaborated in Nelson et al. [24]. Adeno is a type of cancer originating in the glandular tissue of organs such as the lungs, colon, and breast. Meso is a type of cancer that affects the mesothelial cells that line the pleural cavity (the lining of the lungs) and other organs. It is most commonly caused by exposure to asbestos. If not treated early, Adeno can spread to nearby lymph nodes and other organs, making it more difficult to treat. It is also known to have a higher rate of recurrence. Meso is particularly dangerous because it is often not diagnosed until it has advanced to later stages. It can take decades for symptoms to appear after initial asbestos exposure. Additionally, the treatment options for Meso are limited, and the prognosis is generally poor. Adeno and Meso are serious diseases, and prompt diagnosis and treatment are essential for the best possible outcome.

With the advent of machine learning algorithms, such as artificial neural networks (ANN) and deep learning algorithms, the classification performance of Adeno and Meso is improved, as discussed in Podolsky et al. [25]. It is imperative to note that the selection of machine learning algorithm and the specific gene expression data used to train the model can greatly impact the classifier's performance. Additionally, the availability of labelled samples is crucial for training and evaluating the classifier's performance. But as discussed before, nonlinearity, non-gaussian nature, and outlier problems reduce classification accuracy. The Vapnik-Chervonenkis (VC) theory introduced by Vapnik [26] provided a better mathematical framework for improving the generalisation capability of machine learning algorithms. The VC theory solves the problems of nonlinearity, non-Gaussian nature, and outliers in the following way. The VC enables the raw data to map into a high-dimensional feature space to become linearly separable, thus solving the nonlinearity problem. The VC theory assumes data to be in any arbitrary distribution and makes it possible to apply even with data distributed under non-gaussian nature. Also, VC theory is robust to outliers because it only considers the maximal margins between the data points and the decision boundary; therefore, outliers do not significantly impact the decision boundary, making the classifier more robust to noise and outliers. Overall, it is worth mentioning that this area is still under active research, and new methods and techniques are continuously being developed.

Considering all the above aspects, we utilise Hilbert Transform (HT), Detrend Fluctuation Analysis (DFA) and Least Square Linear Regression (LSLR) for DR of the lung cancer-based MGED. FSEL is also performed using Elephant Herd Optimization (EHO) and Cuckoo Search (CS) optimization techniques after Dimensionality Reduction Technique (DRT) to enhance the classifier performance. The classification of lung cancer is performed using Bayesian Linear Discriminant Classifier (BLDC), Naive Bayes Classifier (NBC), Random Forest (RF), Decision Tree (DT), and Support Vector Machine (SVM). The SVM is further explored with Linear, Polynomial and Radial Basis Function (RBF) kernels. The classifier's performance is analysed both with and without FSEL methods. The paper is organised in the following way. Section 2 explains the materials and methods. Section 3 discusses the methodology for DR and FSEL. The classifiers are discussed in section 4 of the paper. The results are discussed in section 5. Section 6 concludes the paper.

## 2 Materials and methods

**Fig. 2:**
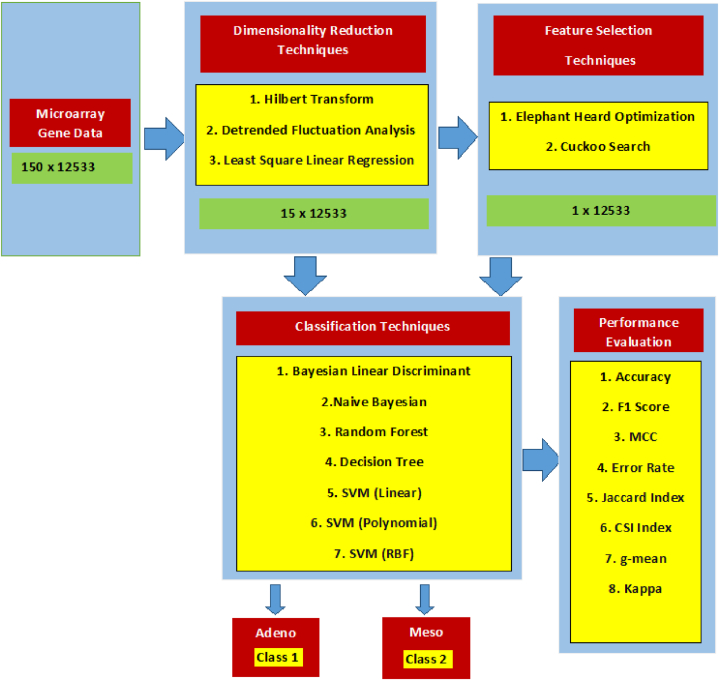
Methodology for DR and Classification for MGED for lung cancer.

The overall methodology of this research work is summarized in Fig. 2. First, DR is performed on the MGED to reduce the data complexity while retaining important information. This step enables easy visualization and analysis of data. After DR, we choose two approaches to proceed further. In the first approach, after DR, using classification techniques, the gene array is classified into two classes Adeno and Meso. The classifier performance is then analysed through the various performance metrics. The second approach adopts Meta heuristic FSEL techniques after DR to find the relevant features. Now based on the reduced set of selected features, the classification techniques will classify the MGED Class 1 and Class 2. As done previously, comparing and contrasting the Meta heuristic FSEL aided classification is performed using the same metrics.

### 2.1 Description of the dataset

The implemented research uses Lung Harvard 2 (LH2) dataset containing MGE [27]. The dataset utilised in this research is publicly available at (Link: https://leo.ugr.es/elvira/DBCRepository/LungCancer/LungCancer-Harvard2.html). This dataset is commonly used in computer vision and machine learning to classify lung cancer. It consists of CT scans of patients' chests and associated labels indicating whether or not they have lung cancer. The images in the dataset are pre-processed to extract lung regions and are typically resampled to a common resolution for ease of processing. The dataset includes a large number of samples with high variability in the appearance of lung nodules under the presence of both benign and malignant cases. So, it is widely used in training and assessing various methodologies for lung cancer classification and is considered a benchmark in the field. As mentioned in Gordon et al. [27], the dataset comprises two classes: Adeno and Meso. Of the 181 samples, 150 are Adeno, and 31 are malignant Meso. 12533 genes describe each of the tissue sample. The rows in the LH2 dataset is 12534, The final row indicates the class labels: ADCA for Adeno and MPM for Meso. The class imbalance problem is addressed in the following way. The 150 tissue samples of adeno lung cancer data are considered model I and the remaining 31 meso lung cancer data samples are considered model II. The 10-fold cross-validation technique is employed for both models. The model I output is acquired by furnishing the test data multiple times, and based on the acquired output, a sample class is assigned. The output of model I is denoted by A0. Similarly, the process is repeated for model II, and M0 denotes the obtained output. The argmax(A0, M0) accurately measures classification, which overcomes the class imbalance problem of the lung cancer dataset.

MGED usually comprises expression levels of large number of genes, making a high-dimensional dataset. The enormous amount of features makes data analysis cumbersome and envisage the interactions among the genes. DR is suitable in these situations to reduce the amount of features while conserving the significant data. So it is easier to identify patterns and associations in MGE to improve the outcome of machine learning algorithms and classification. Feature extraction combined with DR generates new features that are constructive and significant for classification. The goal of feature extraction is to categorize and bring out the pertinent information from the raw data to be used as input features for a machine learning framework. Thus, DR can also be viewed as feature extraction, as it extracts the most important information from the raw data and represents it in a lower-dimensional space. However, DR focuses on reducing the number of features, while feature extraction focuses on creating new, more informative features.

## 3 Dimensionality Reduction Techniques

DR is a critical step in analyzing MGED. The curse of dimensionality arises in MGED because the dataset typically consists of measurements for thousands of genes across a relatively small number of samples. The curse of dimensionality can also lead to challenges such as increased computational complexity, increased risk of overfitting, and decreased interpretability of results. DRT help alleviate these challenges by reducing the number of features while preserving relevant information. Microarray data often contains noise, measurement errors, and redundant or irrelevant features. These factors can obscure the underlying patterns and relationships within the data. By reducing the dimensionality, we can filter out noisy and redundant features, focusing on the most informative and discriminative ones. This helps improve the signal-to-noise ratio, leading to more robust and accurate analysis results. High-dimensional data requires more computational resources and storage space for processing and analysis. DR reduces the data's dimensionality, making subsequent analysis tasks more computationally efficient and requiring less memory. This efficiency is especially important for large-scale microarray datasets, as it enables faster analysis and facilitates the scalability of algorithms.

For improving classification performance, it is important to find a subset of features with enhanced discriminatory power. The discrimination power is significantly impacted when the data is higher dimensional. So, implementing methods to transform the high-dimensional data to lower-dimensional data keeping the meaning and characteristics of the data. This method is called DR. The DR is more accurate than other estimation techniques. So estimation model is simpler even in noisy data as DR is fully a mathematically driven method.

The DR technique also delivers a smoother version of the raw data by a simple convolution with the kernel.

### 3.1 Hilbert Transform

The HT DRT is a non-linear method for reducing the dimensionality of high-dimensional data by mapping it to a lower dimensional space. The technique uses HT mathematical operators to analyse time domain signals. Fang et al. [28] proposed a non-linear DRT based on the HT. Using the HT, the method first maps the high-dimensional data to a feature space. Then it applies singular value decomposition and principal component analysis to reduce the dimensionality of the transformed data. The method is evaluated on several benchmark datasets and compared with other non-linear DRT. The results showed that the HT method preserves the non-linear data structures with more meaningful lower-dimensional representations.

The HT function given as Fang et al. [28] is represented in (1)

(1) $$h\left(t\right)=\frac{1}{\left(\pi t\right)}$$

The convolution of the signal g(t) with h(t) is considered as the HT of ‘g’ is represented in (2)

(2) $$g^{ˆ}\left(t\right)=\int_{-\infty }^{\infty }g\left(\tau \right)\;h\left(t-\tau \right)\;d\tau$$

From (1), $g^{ˆ}\left(t\right)$ is expressed as (3)

(3) $$g^{ˆ}\left(t\right)=\frac{1}{\pi }\int_{-\infty }^{\infty }\frac{g\left(\tau \right)}{t-\tau }d\tau$$

### 3.2 Detrend Fluctuation Analysis (DFA)

DFA is another method for DR. DFA uses a time-series analysis technique to quantify long-range correlations in time-series data. The method first detrends the time-series representation of the data by removing the trends, then calculates the fluctuations of the residuals, and finally applies DRT such as singular value decomposition or principal component analysis to the fluctuations. Peng et al. [29] show how DFA can identify complex systems and distinguish them from random or periodic systems. The authors also demonstrated DFA's utility to various real-world systems, such as heart rate variability, physiological signals, and financial time series. The results show that DFA provides a robust and sensitive measure of long-range correlations in complex systems and can be used for characterising and comparing different systems. This process can also preserve important non-linear structures in the data and produce a more meaningful lower-dimensional representation.

DFA is a statistical method for quantifying long-range correlations and self-similarity in time series data. It involves detrending the original time series to eliminate trends and fluctuations and then calculating the residuals' root mean square (RMS) fluctuation as a function of the time scale. DFA can be used as a DRT to transform a high-dimensional time series into a one-dimensional representation that captures the underlying scaling behaviour. The resulting scaling exponent obtained from the DFA can then be used as a feature to describe the time series data, as mentioned in Peng et al. [29].

For a given time series of data x(i), i = 1, 2, …, N, the DFA proceeds through the following steps.

First, the time series data is integrated by summing all x(i) values to generate a new time series y(i) as given in (4)

(4) $$y\left(i\right)=\sum_{j=1}^{i}x\left(j\right)$$

Now detrending is performed on y(i) by fitting an appropriate polynomial function that minimises the least square error between F(i) and y(i).The residual between y(i) and F(i) is represented by (5)

(5) r(i)=y(i)−F(i)

Now scaling is performed by dividing the time series into L non-overlapping segments of equal length. For each segment, the RMS fluctuation of the residuals is calculated as given in (6)

(6) $$F\left(s\right)=\sqrt{\frac{1}{L}\;*\sum_{i=1}^{L}{r\left(i\right)}^{2}}$$

Here ‘s’ is the time scale, defined as the segment length.

Now, the scaling exponent α is then calculated by taking the logarithm of F(s) and plotting it against log(s), resulting in a power-law relationship of the form as given in (7)

(7) log(F(s))=αlog(s)+constant.

The slope of the log-log plot, α, is the scaling exponent that characterizes the long-range correlations and self-similarity in the time series.

### 3.3 Least Square Linear Regression (LSLR)

Another important method for DR is the LSLR. This concept was first introduced by Hotelling [30], who used principal component analysis (PCA) as a tool for regression analysis. Using PCA, the LSLR technique transforms the high-dimensional data into a lower-dimensional space and then applies a linear regression model to the transformed data. Here, the mapping is obtained by minimising the sum of squared errors among the Dimensionally Reduced (DimRed) representation and the raw data. The resulting mapping can transform the large-dimensional data into a lower-dimensional space. Thus PCA is a linear DRT that finds a low-dimensional representation of the large-dimensional data by identifying the directions of maximum variance. LSLR, as mentioned in Hastie et al. [31], performs DR by finding the best-fit line that portrays the association between the independent variables (features) and the dependent variable (target). Consider a set of N observations of the form (x_1_, y_1_), (x_2_, y_2_), …, (x_N_, y_N_), where x_i_ is the ith observation of the independent variables and y_i_ is the corresponding observation of the target variable. Then as elaborated in Hastie et al. [31], the LSR solution is a linear equation of the form as given in (8)

(8) $$y=\beta_{0}+\beta_{1}x_{1}+\beta_{2}x_{2}+\ldots +\beta_{p}x_{p}$$

Where β_0_, β_1_, β_2_, …, β_p_ are the parameters of the linear model, and p is the number of independent variables. The goal of LSR is to find the values of the parameters that minimise the sum of squared differences between the original and forecasted values of the target variable., defined as in (9)

(9) $$SSE=\sum_{i=1}^{N}{\left(y_{i}\;-\;\left(\beta_{0}+\beta_{1}x_{1}+\beta_{2}x_{2}+........+\beta_{P}x_{p}\right)\right)}^{2}$$

The solution to the LSLR problem is obtained by finding the values of the parameters that minimise SSE. After the DRT on MGED and the subsequent results are examined by the statistical parameters such as mean, variance, skewness, kurtosis, Pearson Correlation Coefficient (PCC), f-test, T-test, p-value, and CCA to reveal whether the DimRed data is contains the significant patterns and properties of the MGED in the reduced subspace.

Table 1 contains the various statistical features for three types of Adeno and Meso cases of MGED after DR. As displayed in Table 1, the HT and DFA-based DRT display large mean and variance between the classes. But the LSLR DRT shows a small mean and variance deviations, representing clustered data inside the cancer classes. All the DRT methods exhibited positive skewness and flat kurtosis. PCC designate the great correlation inside the output classes. The F-test, T-test and p-value are insignificant for the DR outputs of the MGED. This designates that the values are neither Gaussian nor linear. The results obtained by investigating histogram, Normal Probability Plots (NPP) and scatter plots of DRT outputs underlines the statistical observations properties of MGED like nonlinearity, scattering, and non-Gaussian. Canonical correlation Analysis (CCA) picturizes the correlation between DRT outcomes between the Adeno and Meso classes. The small CCA in Table 1 indicate that the DR results are least correlated between the two classes.

**Table 1:** Average statistical features for DimRed adeno and meso cases.

Sl.No	Statistical Features	DRT					
		HT		DFA		LSLR	
		Adeno	Meso	Adeno	Meso	Adeno	Meso
1	Mean	94.16982	100.2762	53.32597	68.57455	0.001254	0.001567
2	Variance	79474.82	124963.2	23974.98	46429.21	0.000539	0.000608
3	Skewness	8.919005	10.22995	12.23451	13.9916	22.48083	25.39857
4	Kurtosis	110.3538	145.497	267.0666	319.4125	803.6333	1013.376
5	PCC	0.936883	0.952273	0.813286	0.880523	0.61776	0.725884
6	F-test	0.03997	5.8938E-110	0.005032	2.6914E-143	0.001474	1.0305E-303
7	T-test	0.100605	0.053942	0.000267	7.27E-20	0.106677	3.83E-06
8	p-value <0.01	0.460647	0 0.480202	0.499922	0.5	0.4532	0.5
9	CCA	0.3704		0.2689		0.2318	

**Fig. 3:**
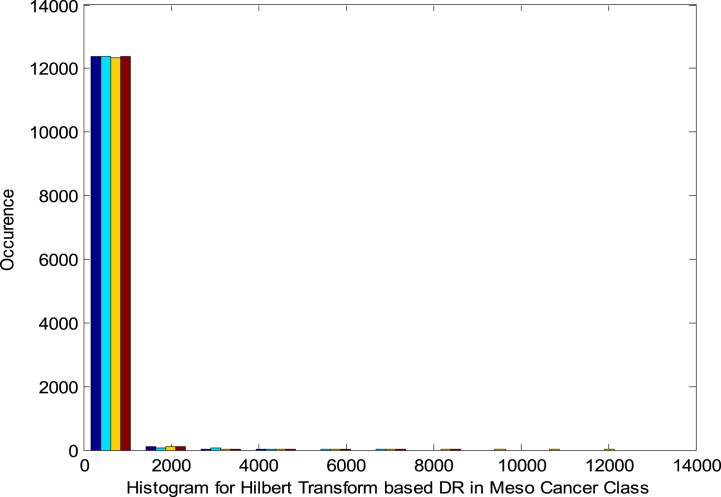
Histogram for HT based DRT in Meso Class.

Fig. 3 shows the Histogram for HT based DRT in Meso Class. It is identified from Fig. 3 that the histogram is non-linear and extremely skewed. So non-linearity of MGED is retained even after the DR. This histogram also displays a non-Gaussian property of the DRT.

**Fig. 4:**
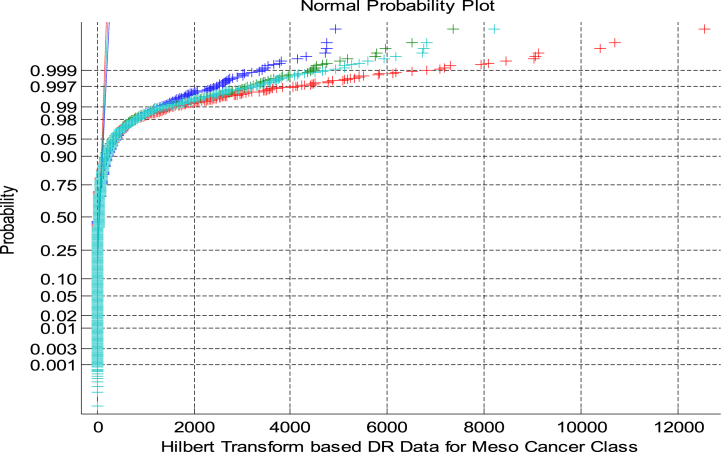
NPP for HT based DRT in Meso Class.

Fig. 4 shows the NPP for HT based DRT in Meso Class. As from Fig. 4 shows that the NPP for the HT indicates the non-linear clustered and non-Gaussian nature of the DRT.

**Fig. 5:**
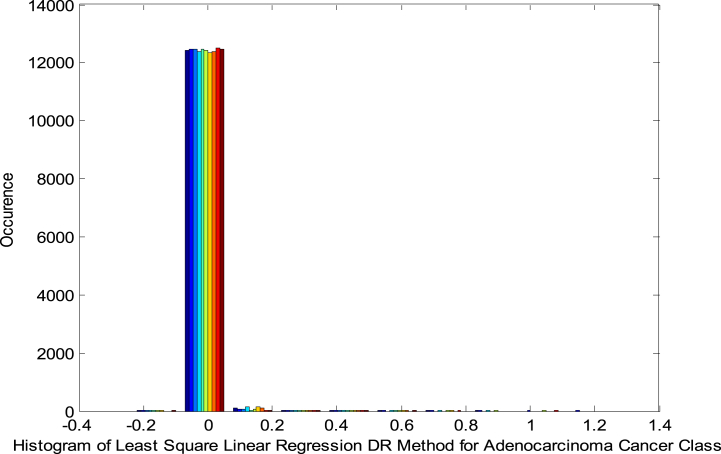
Histogram of LSLR DRT in adeno class.

Fig. 5 presents the Histogram of the LSLR DRT in Adeno Class. As shown in Fig. 5, the histogram indicates a shift in the plot, representing the non-linear nature of the DRT.

**Fig. 6:**
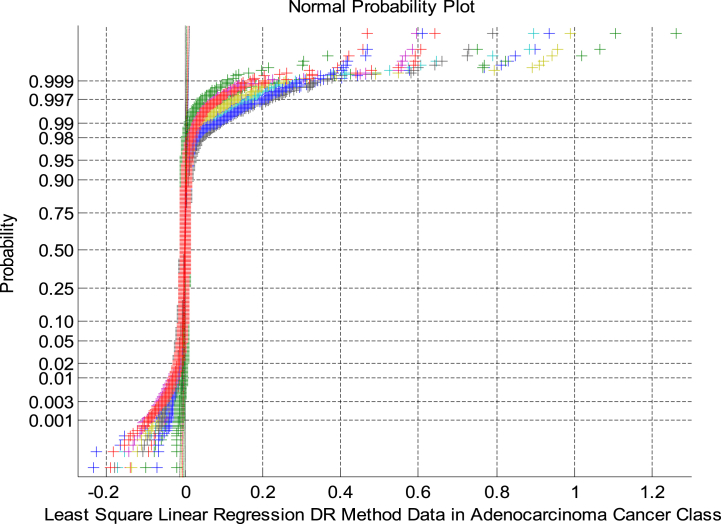
NPP for LSLR based DRT in Adeno Class.

Fig. 6 exhibits the NPP of the LSLR DRT in Adeno Class. As shown in Fig. 6 that the NPP indicates the non-linearity and non-Gaussian behaviour of the DR outputs.

**Fig. 7:**
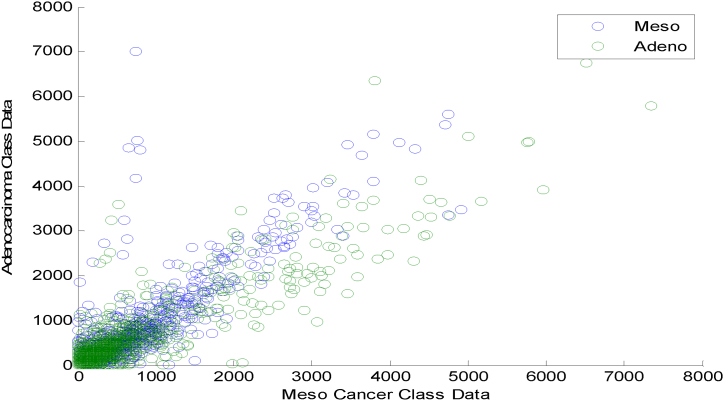
Scatter plot for HT based DRT in Meso and Adeno Classes.

The scatter plots exhibit the non-Gaussian distribution of the variables under analysis. Fig. 7 shows a Scatter plot for HT based DRT in Meso and Adeno Classes. It is observed that Fig. 7 displays a sparse, overlapped and non-Gaussian nature of the HT DRT among the two cancer classes.

**Fig. 8:**
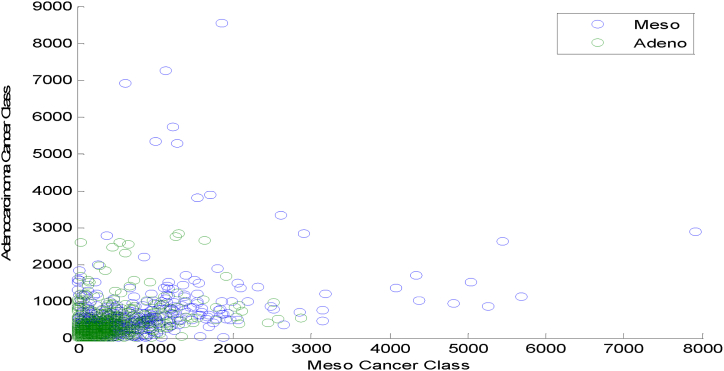
Scatter plot for DFA based DRT in Meso and Adeno Classes.

Fig. 8 explores the Scatter plot for DFA based DRT in Meso and Adeno Classes. Fig. 8 indicates a clustered, Non-linear overlapped, outliers and non-Gaussian nature of the DFA DRT among the two cancer classes.

**Fig. 9:**
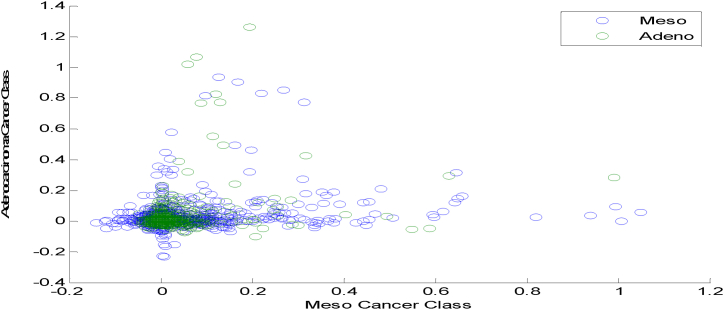
Scatter plot for LSLR Based DRT in Meso and Adeno Classes.

Fig. 9 exhibits the Scatter plot for the LSLR-based DRT in Meso and Adeno Classes. Fig. 9 demonstrates a clustered, Non-linear overlapped, and non-Gaussian nature of the LSLR DRT among the two cancer classes.

### 3.4 Feature selection techniques

Further, we explore the possibilities of FSEL after DRT with the help of meta-heuristic optimization techniques like EHO and CS. The EHO algorithm is a meta-heuristic optimization technique inspired by the collective behaviour of elephants in nature. As elaborated by Wang et al. [32], it can be applied to FSEL from MGED. In this context, EHO can be used to select the most relevant features after DRT. The technique involves evaluating a fitness function for every subset of DimRed features and updating the search mechanism based on the best results gathered from the herd of elephants rather than just one individual. Likewise, CS is a meta-heuristic optimization technique motivated by the behaviour of cuckoo birds in its habitat. As performed by Q. Zhang et al. [33]. CS can be applied to FSEL problems of DimRed relevant features for MGED analysis. Here, CS extracts the relevant features, estimates a fitness function for every subset of DimRed relevant features, and rework on the solution searching mechanism based on the best results gathered from the cuckoos. The FSEL step using EHO and CS leverages computational complexity and enhances the classifier outcomes.

#### 3.4.1 Elephant Herd Optimization (EHO)

EHO is a meta-heuristic optimization algorithm proposed by Wang et al. [34], motivated by the behaviour of elephants in the African regions. The technique is applied in various fields, such as feature selection can effectively solve optimization problems. In FSEL, the goal remains to select a subset of features from a higher dimensional set of the most informative features significant to the target variable. The EHO algorithm uses a herd of elephants to find the best solution. The elephant in the herd represents a candidate solution, and the algorithm uses a blend of global and local search methods to move the elephants towards the best solution. EHO is a promising FSEL technique because it effectively balances global and local searches and handles high-dimensional data.

EHO first initializes the elephant herd with random locations in the feature space. The location of each elephant and the overall movement of the heard, represented by Pham et al. [35] is given by (10)

(10) $${x_{i}}^{new}={x_{i}}^{old}+\alpha \;\left(\;X_{best}-\;{X_{i}}^{old}\right)\;*\;r$$

Where ${x_{i}}^{new}$ and ${X_{i}}^{old}$ are the old and new locations of the elephant, α∈[0,1] is the control variable, r ∈[0,1] is a randomly produced number. Each elephant in the herd has a memory that records its best location in the feature space. The best location is reworked by the expressions given in (11)

(11) $$\begin{array}{c} X_{best}=\beta \;*X_{centre}\\ X_{centre}=\frac{1}{n}\;*\;\sum_{i=1}^{n}x_{i} \end{array}$$

Here $X_{best}$ is the best location of the elephant with the control variable β∈[0,1].

Like the best position, the worst position is also captured and updated as given in (12)

(12) $$X_{worst}=X_{\min}+\left(X_{\max}-X_{\min}+1\;\right)\;*\;rand$$

Here $X_{\min}$ and $X_{\max}$ represents the maximum and minimum values of the search space.

For a population size of 1253 elephants with a max iteration of 1000 and MSE, is the decision criteria for α and β is made through a trial and error technique. The α and β are set at critical values of 0.65 and 0.68, respectively.

#### 3.4.2 Cuckoo Search (CS)

CS is a meta-heuristic optimization algorithm proposed by Yang et al. [36], inspired by the behaviour of cuckoos and their eggs. It is also applied in various fields, such as FSEL, and effectively solves optimization problems. The feature subset is selected by optimizing metrics based on MSE and selecting the features that result in the lowest value of the metric. The algorithm uses a set of cuckoos to explorethe best solution. Each cuckoo represents a candidate solution, and the algorithm uses a combination of randomization and local search methods to move the cuckoos towards the best solution. One of the key features of CS is its use of a Levy flight, a random walk with a heavy-tailed distribution, to control the movement of the cuckoos. This helps to ensure that the cuckoos can explore the feature space efficiently while allowing them to move quickly to promising regions. So, CS is chosen as a FSEL technique due to its capability to balance exploration and exploitation and handle high-dimensional data effectively. The CS algorithm first initializes a set of cuckoos with random positions in the feature space. The position of each cuckoo in the population is updated using expression (13)

(13) $${x_{i}}^{new}={x_{i}}^{old}+\alpha \;*\;\left(r\;-\;0.5\right)\;*\;\left(x_{best}\;-\;x_{old}\right)+\beta *\;\left(r\;-\;0.5\right)\;*\;\left(x_{global\;best}\;-\;x_{old}\right)$$

where ${x_{i}}^{old}$ and ${x_{i}}^{new}$ are the old and new positions of the cuckoo, α and β are parameters that control the influence of the local and global best solutions, $x_{best}$ is the best position of the cuckoo, and $x_{global\;best}$ is the best position of all cuckoos in the population. r ∈[0,1] is randomly generated.

Next, the Levy flight technique is used to control the random movement of the cuckoos. It is defined by (14)

(14) $$s=\sigma \;*\;r^{\frac{-1}{\mu }}$$

where ‘s’ is the step size, σ is a parameter that controls the scale of the Levy flight, and μ is a parameter that controls the shape of the Levy flight.

The best nest in the population is selected based on its fitness value. The fitness value is calculated using an evaluation function MSE, and the best nest is used as the starting point for the next iteration of the algorithm. Also, a fraction of the nests in the population is abandoned and replaced by new cuckoos. This ensures that the population is diversified and that new solutions are explored. For a population of 1253 and a maximum of 1000 iterations, the parameters *p, a*, β, and γ in the CS algorithm are set to 0.4, 1 and 1.3 through trial and error.

## 4 Classification techniques

The classification of lung cancer is the most important objective of this research. So the selection of the classifier is a prime concern. As mentioned by Liu et al. [37], (BLDC) machine learning algorithm is used for efficient classification from MGED.BLDC can handle small sample sizes and high-dimensional data, often in MGED. BLDC can also handle different forms of prior information and incorporate this information into the classification process. This characteristic of BLDC delivers more classification accuracy and improved performance. BLDC can also provide a probabilistic framework for classifying genes, which makes it easy to interpret and understand the results. Lu et al. [38] used Naive Bayesian Classifier (NBC) for gene classification in MGED. NBC is a straightforward algorithm that requires minimal computational resources and is easy to implement. So, NBC can handle big datasets, and hence it is appropriate for MGED which often consists of tens of thousands of genes. NBC is robust to irrelevant or redundant features, making it well-suited for MGED where many genes may not be relevant for the classification task. Like BLDC, NBC also provides a probabilistic framework for classifying genes.

As mentioned in Breiman [39], Random Forest Classifier (RFC) is a decision-tree-based machine learning algorithm that can be used for gene classification in MGED. RFC achieves high classification accuracy, especially for datasets with complex relationships between features. RFC is robust to noisy data and irrelevant features, making it well-suited for MGED where many genes may not be relevant for the classification task. Although the internal workings of a RF can be complex, the algorithm's output can still be easily interpreted and understood. RFC can also provide information about the relative importance of each feature, which can be useful for FSEL and interpretation of the results. A Decision Tree Classifier (DTC) is a machine learning algorithm that can be used for gene expression data classification in microarray experiments performed by Gharehgozli et al. [40]. The algorithm builds a tree-like structure to make decisions based on the characteristics of the data. DT is easy to understand and interpret as they represent the decision process clearly and concisely. They can handle missing values without the need for imputation. DT can capture non-linear relationships between features and target variables and handle numeric and categorical variables, making them versatile gene classification algorithms. DTs require very little data pre-processing compared to other algorithms, making them a fast and efficient option for gene classification.

The VC theory significantly impacts the development of SVMs and kernel methods. So, SVM have become a popular machine learning algorithm that can be utilised for gene expression data classification in microarray experiments, as mentioned in Cruz et al. [41]. The algorithm finds the boundary that separates the different classes in the data. SVM delivers high accuracy in classification tasks, making it a popular choice for gene classification in microarray experiments. SVM algorithms are particularly useful for handling high-dimensional data, often in gene expression analysis. SVM algorithms are less prone to overfitting than other algorithms, making them a more reliable option for gene classification. The kernel trick in SVM algorithms allows for non-linear boundary decisions, making it capable of capturing complex relationships in the data. SVM algorithms can be used for binary and multi-class classification problems, making them a versatile option for gene classification.

### 4.1 Bayesian Linear Discriminant Classifier (BLDC)

BLDC is a method based on Bayesian statistical principles. The basic idea behind BLDC is to apply the Bayes theorem to find the posterior probabilities of each class given the observed features. Bayes theorem as given by Bishop et al. [42] is expressed in (15)

(15) P(y|x)=(P(x|y)*P(y))/P(x)

where P(y|x) is the posterior probability of class y given the observed features x, P(x|y) is the likelihood of observing the features x given the class y, P(y) is the prior probability of class y, and P(x) is the marginal likelihood of observing the features x.

The mean and covariance of each class are estimated from the training data. Let x_i_ be the ith feature vector and y_i_ be the class label. The mean of class y is given by (16)

(16) $$\mu_{y}=\left(\frac{1}{N_{y}}\right)\;*\sum_{i}^{y_{i}=y}x_{i}$$

Here $N_{y}$ is the number of feature vectors in class y. The covariance of class y is given by (17)

(17) $$\sigma_{y}=\left(\frac{1}{N_{y}}\right)\;*\sum_{i}^{y_{i}=y}\left(x_{i}-\mu_{y}\right)\;{\left(x_{i}-\mu_{y}\right)}^{T}$$

The posterior probabilities of each class are used to define a discriminant function for each class. The discriminant function for class y is given by (18)

(18) $$g_{y}\left(x\right)=-0.5*\;\log|\sigma_{y}|\;-\;0.5\;*\;{\left(x-\mu_{y}\right)}^{T}*{\sigma_{y}}^{-1}*\left(x-\mu_{y}\right)+\log\;\left(P\left(y\right)\right)$$

where | $\sigma_{y}$ | is the determinant of the covariance matrix $\sigma_{y}$ and log(P(y)) is the log of the prior probability of class y. To make a prediction for a new feature vector x, the discriminant functions for every class is assessed and the class with the maximum value is nominated.

### 4.2 Naive Bayesian Classifier (NBC)

The NBC is a simple and effective algorithm for classification based on Bayesian statistical principles. The following are key equations and expressions used in the Naive Bayesian algorithm by Bishop et al. [42].

The prior probability of each class is estimated from the training data. Let y_i_ be the class label of the ith training instance and N be the total training occurrences. The prior probability of class y is given by (19)

(19) P(y)=(numberofinstancesofclassy)/N

The likelihood of observing the features x given the class y is assumed to be the product of the individual probabilities of each feature. Let (x_i_,j) be the jth feature of the ith training instance. The likelihood of class y is given by (20)

(20) $$P\left(x|y\right)=P\left(x_{1},y\right)\;*\;\;P\left(x_{2},y\right)\;*\;\ldots \;*\;P\left(x_{n},y\right)$$

Here n is the number of features and P(x_j_,y) is the probability of observing the jth feature given the class y. The posterior probability of each class given the observed features x is found using Bayes theorem as given by (21)

(21) P(y|x)=(P(x|y)*P(y))/P(x)

To make a prediction for a new feature vector x, the posterior probabilities for every class are estimated, and the class with the maximum probability is chosen.

### 4.3 Decision Tree Classifier (DTC)

A DT is a tree-like model used for classification and regression tasks that makes predictions by recursively splitting the data into smaller subsets based on feature values. The prediction for a new instance is achieved by traversing the tree from the root node to a leaf node, where the class label is stored. At each node in the tree, a feature and a threshold are chosen to split the data into two subsets. The impurity of a node is a measure of how “mixed” the class labels are in the node. Common impurity measures for classification tasks include Gini impurity and information gain (entropy).

The Gini impurity, as given by Hastie et al. [43], is given in (22)

(22) $$Gini\;\left(p\right)=1-\sum_{i=1}^{C}{\left(p_{i}\right)}^{2}$$

where p is the proportion of instances of class i in the node, and C is the number of classes.

The expression for information gain is given by (23)

(23) $$Information\;Gain\left(S,A\right)=Entropy\;\left(S\right)\;-\;\sum_{\nu \in values\left(A\right)}\frac{\left|S_{v}\right|}{\left|S\right|}*Entopy\left(S_{\nu }\right)$$

where S is the set of instances in the node, A is a feature, S_***ν***_ is the subset of occurrences with the value ***ν*** of feature A, and values (A) are the possible values of feature A. The feature and threshold that result in the maximum reduction in impurity are chosen as the best split. The best split is given by (24)

(24) $$BestSplit\left(S\right)={\mathrm{argmax}}_{a,t}ImpurityMeasure\left(S\right)-\sum_{\nu \in values\left(A\right)}\frac{\left|S_{v}\right|}{\left|S\right|}*ImpurityMeasure\;\left(S_{\nu }\right)$$

where S is the set of instances in the node, A is a feature, t is a threshold, and S_v_ is the subset of instances with the value v of feature A. The final prediction for a new instance is made by traversing the tree from the root node to a leaf node, where the class label is stored.

### 4.4 Random Forest Classifier (RFC)

In a RFC problem, each DT in the forest outputs a class label for a given input. The final prediction is made by majority voting among all the trees. A DT is a tree-like model that makes predictions by recursively splitting the data into smaller subsets based on feature values. The prediction for a new instance is made by traversing the tree from the root node to a leaf node, where the class label is stored. The DT in RF is built using a greedy algorithm that splits the data at each node based on the feature that provides the maximum reduction in impurity, where the impurity is usually measured using Gini impurity or entropy. The algorithm stops when a stopping criterion is met, such as reaching a maximum depth or having a minimum number of samples at a leaf node. At each node in the DT, a random subset of features is selected to choose the split. In RF, the training data is first randomly sampled with replacement to create multiple bootstrapped samples. Each DT is then trained on one of the bootstrapped samples. The random subset of the data and features is drawn using bootstrapping and random subspace sampling, respectively. In bootstrapping, a sample of size N (the raw data size) is drawn with a replacement from the raw data. In random subspace sampling, a random subset of the features is selected at each split of the DT. The final prediction for a new occurrence is made by combining the predictions of all the DT in the forest. A common aggregation method is to take the majority vote on the class labels predicted by each tree.

The equation for prediction using the majority vote in RF as defined by James et al. [44] is given by (25)

(25) $$y={\mathrm{argmax}}_{c}\sum_{t=1}^{T}y_{t}=c$$

where y is the final prediction, T is the number of DT in the forest, y_t_ is the prediction of the tth tree, and c is a class label.

### 4.5 Support vector machine classifier (linear)

SVM is a powerful machine learning algorithm for classification and regression tasks. The linear SVM is a variant of SVM used for linearly separable data. The objective of the linear SVM is to find a hyperplane that separates the data into two classes with the maximum margin, where margin is defined as the distance between the hyperplane and the closest data points, called support vectors.

The equation for the objective function of the linear SVM is represented as (26)

(26) $${minimize}_{w,b}\frac{1}{2}{\left\|w\right\|}^{2}+C\sum_{i=1}^{N}y_{i}\;\left(w^{t}x_{i}+b\right)<=1$$

Here w is the weight vector, b is the bias, x_i_ is the ith instance, y_i_ is the class label of the ith instance, C is the regularisation parameter, and N is the number of instances. Given a new instance, its prediction is made by calculating the value of the decision function.

The equation for the decision function is represented as (27)

(27) $$f\left(x\right)=sign\;\left(w^{T}\;x+b\right)$$

Here x is the new instance, w is the weight vector, and b is the bias. The sign function returns +1 if the value is positive and −1 if the value is negative, indicating the predicted class label.

### 4.6 Support vector machine classifier (polynomial)

The polynomial SVM is a variant of SVM used for non-linearly separable data by converting the features into a larger dimensional space and finding a linear separator in that space. The polynomial SVM transforms the original features into a larger dimensional space utilising polynomial mapping. The degree of the polynomial governs the number of dimensions in the transformed space.

The expression for the feature transformation is represented as (28)

(28) $$x^{\prime }=\left(x_{1},x_{2},...,x_{d},x_{1}^{2},x_{1}x_{2},...,x_{d}^{2},...,x_{1}^{d},x_{2}^{d},...,x_{d}^{d}\right)$$

Here x is the original feature vector, x' is the transformed feature vector, and d is the degree of the polynomial. The target function of the polynomial SVM is the same as that of the linear SVM, which is to find a hyperplane that separates the data into two classes with the maximum margin.

The equation for the target function of the polynomial SVM is represented by (29)

(29) $${minimize}_{w,b}\frac{1}{2}{\left\|w\right\|}^{2}+C\sum_{i=1}^{N}y_{i}\;\left(w^{t}x_{i}^{\prime }+b\right)<=1$$

The decision function accordingly changes to (30)

(30) $$f\left(x\right)=sign\;\left(w^{T}x^{\prime }+b\right)$$

### 4.7 Support vector machine classifier (RBF)

The Radial Basis kernel Function (RBF) SVM is a variant of SVM used for non-linearly separable data by transforming the features into a larger higher dimensional space using a radial basis kernel function and finding a linear separator in that space. The RBF maps the original features into a larger dimensional space by computing a nonlinear function of the Euclidean distance between two instances. The expression for the RBF is given by (31)

(31) $$K\left(x_{i},x_{j}\right)=e^{\left(-\gamma {\left\|x_{i}-x_{j}\right\|}^{2}\right)}$$

where x_i_ and x_j_ are the feature vectors of instances i and j, gamma is a parameter that regulates the width of the RBF, and ||.|| is the Euclidean distance. The target function of the SVM RBF is the same as that of the linear SVM, which is to find a hyperplane that separates the data into two classes with the maximum margin.

The target function of the SVM RBF is given by (32)

(32) $$\begin{array}{c} {minimize}_{a}\frac{1}{2}\sum_{i=1}^{N}\sum_{j=1}^{N}a_{i}{\;a}_{j}y_{i}y_{j}\;K\left(x_{i}x_{j}\right)-\sum_{i=1}^{N}a_{i}\\ \begin{array}{c} subject\;to:\\ \sum_{i=1}^{N}a_{i}y_{i}=0 \end{array}\\ 0<=a_{i}<=Cfor\;i=1,2,...,N \end{array}$$

Here a is the dual vector, x_i_ is the ith instance, y_i_ is the class label of the ith instance, C is the regularisation parameter, and N is the number of instances. Given a new instance, its prediction is performed by calculating the value of the decision function.

The expression for the decision function is represented by (33)

(33) $$f\left(x\right)=sign\;\left(\sum_{i=1}^{N}a_{i}y_{i}\;K\left(x_{i},x\right)+b\right)$$

where x is the new instance, a is the dual vector, y_i_ is the class label of the ith instance, $K\left(x_{i},x\right)$ is the RBF kernel, and b is the bias.

### 4.8 Training and testing of classifiers

The dataset has limited training data, so we utilised k-fold cross-validation to estimate the performance of the machine-learning model. This popular method, described by Fushiki et al. [45], divides the dataset into k equally sized subsets, or “folds.” The model is trained on all the data except the ith fold and then tested on that fold. This methodology is repeated for all k folds. The average performance estimates from the k folds is computed to provide an overall estimate of the model's performance. After training and validating the model using k-fold cross-validation, it can be retrained on the full dataset to predict new, unseen data. Using 10-fold cross-validation, we ensure a more reliable performance estimate than a simple train-test split, as it utilizes all the raw data.

The research obtained 1253 DimRed features per patient from the LH2 dataset and the training of classifiers was performed in multiple trials. Cross-validation was utilised to eliminate dependency on the test set pattern choice. The training procedure is monitored by overseeing the Mean Square Error (MSE), which is defined by Wang et al. [46] is given in (34)

(34) $$MSE=\frac{1}{N}\sum_{j=1}^{N}{\left(O_{j}-T_{j}\right)}^{2}$$

Where Oj- is the observed value and T_j_ is the target value of the model at time j;

j = 1 and 2, and N is the total number of observations per epoch. In this research it is 1253. As the training progress the MSE value touched at 1.0 E−10 within 1000 iterations.

The parameters in confusion matrix as given in Table 2 is expressed as. True Positive (TP): A subject is correctly branded as Adeno True Negative (TN): A subject is correctly branded as Meso.

**Table 2:** Confusion matrix for Lung Cancer Detection.

Truth of Clinical Situation		Predicted Values	
		Adeno	Meso
Actual Values	Adeno	TP	FN
	Meso	FP	TN

False Positive (FP): A subject is incorrectly branded as Adeno instead of Meso.

False Negative (FN): A subject is incorrectly branded as Meso instead of Adeno.

### 4.9 Selection of target

The T_j_ for the Meso $\left(T_{Meso}\right)$ is considered nearer to the lower bounds of (0,1) and this target mapping follows the constraint given by (35)

(35) $$\frac{1}{N}\sum_{i=1}^{N}\mu_{i}\leq T_{Meso}$$

where $\mu_{i}$ is the mean feature vectors for the Meso case taken for classification. Similarly, the target value for the Adeno case ($T_{Adeno}$) is is considered nearer to the upper bounds of (0, 1) and this target mapping follows the constraint given by (36)

(36) $$\frac{1}{M}\sum_{j=1}^{N}\mu_{j}\leq T_{Adeno}$$

where $\mu_{j}$ is the average M feature vectors pertaining to Adeno and considered for classification. The consideration is that $T_{Adeno}$ is always above the average value of $\mu_{i}$ and $\mu_{j}$. Also, the deviation between $T_{Adeno}$ and $T_{Meso}$ must follow the constraint given by (37)

(37) $$\|T_{Adeno}-T_{Meso}\|\geq 0.5$$

Considering the constraints (35-37), the targets $T_{Meso}$ and $T_{Adeno}$ for Meso and Adeno classes are selected at 0.1 and 0.95 respectively. After choosing the target, the MSE is utilised for estimating the outcomes of the considered machine learning models.

Table 3 displays the optimum parameters considered for the classifiers during the training processing with trial and error method. The maximum iteration fixed at 1000 for controlling the Convergence Criteria (CC) that is MSE.

**Table 3:** Selection of parameters for classifiers.

Classifiers	Description
BLDC	Prior probability = 0.5, Class mean = 0.85, 0.1; CC = MSE
NBC	Smoothing factor (α) = 0.01, Prior Probabilities = 0.2
RFC	Number of trees:100, Maximum depth of the trees:10, Bootstrap samples:25, Class Weights (CW) = 0.4
DTC	Maximum depth of the tree: 10, Impurity criterion: MSE, CW = 0.45
SVM (Linear)	C (Regularisation Parameter) = 0.85, CW = 0.4, CC = MSE
SVM (Polynomial)	C = 0.76, Coefficient of the kernel function (γ) = 10, CW = 0.5, CC = MSE
SVM (RBF)	C = 1, γ = 100, CW = 0.86, CC = MSE

Table 4 demonstrates the Testing MSE outcomes of the classifiers with and without FSEL (EHO and CS) techniques for three different DRT. The NBC with DFA DRT without FSEL achieved lower testing MSE of 3.61E-06. This low-testing MSE indicates the high accuracy of the classifier. The SVM (Linear) Classifier is positioned with low testing MSE Value of 2.5E-07 for the DFA DRT with EHO FSEL. For CS FSEL, the SVM (RBF) ascertained a low Testing MSE value of 8.1E-07 for both the DFA and LSLR DRT. As indicated by Table 4, the large testing MSE represents the inferior results of the classifier, regardless of the FSEL techniques.

**Table 4:** Classifiers performance for testing MSE without and with FSEL for various DRT.

Classifiers	Without FSEL			With EHO FSEL			With CS FSEL		
	HT	DFA	LSLR	HT	DFA	LSLR	HT	DFA	LSLR
BLDC	1.369E-05	0.000156	3.14E-05	3.721E-05	1.68E-05	7.57E-05	7.84E-06	2.6E-05	0.000169
NBC	8.1E-05	3.61E-06	0.000289	6.76E-06	0.000196	5.63E-05	4.356E-05	3.6E-05	2.4E-05
RFC	7.225E-05	4.1E-05	9.03E-05	4.624E-05	1.96E-06	0.000001	4.624E-05	3.14E-05	4.62E-05
DTC	7.84E-06	8.65E-05	7.74E-05	1.369E-05	8.1E-07	2.25E-06	7.29E-06	4.9E-07	7.92E-05
SVM(Linear)	3.025E-05	4.21E-06	8.41E-06	0.000289	2.5E-07	1.21E-06	2.025E-05	2.56E-06	8.84E-05
SVM(Poly)	6.084E-05	5.33E-05	4.76E-05	0.000169	3.6E-05	7.06E-05	3.969E-05	0.000016	7.23E-05
SVM(RBF)	5.041E-05	9.22E-05	1.76E-05	0.00015625	1.02E-05	1.69E-06	3.721E-05	8.1E-07	8.1E-07

## 5 Results and discussion

The research incorporates classic ten-fold testing and training, where 10 % of input features are utilised for testing and 90 % for training. The selection of performance metrics plays a crucial role in assessing classifier outcome. The confusion matrix is applied to assess the performance of machine learning algorithms, particularly in binary classification, such as separating between malignant and benign cases in lung cancer detection. It enables the calculation of various performance metrics: Accuracy, F1 score, MCC, Error Rate, Jaccard Index, Classification success Index, g-Mean, and Kappa, which are typically used to assess the overall performance of the model. The performance metrics is presented in Table 5.

**Table 5:** Classifier performance metrics.

Performance Metrics	Description of Metrics	Derived from Confusion matrix
Accuracy (Acc)	The average of the number of samples, are identified as positive to number of negative samples	$Accuracy=\frac{\left(TN+TP\right)}{\left(TN+FN+TP+FP\right)}$
F1 Score (F1)	A measure of precision and recall for determining classification accuracy	$F1=\frac{2*TP}{\left(2*TP+FP+FN\right)}$
Mathews Correlation Coefficient (MCC)	Correlation factor for reliability prediction	$MCC\frac{\left(TP*TN-FP*FN\right)}{\sqrt{\left(\left(TP+FP\right)*\left(TN+FP\right)*\left(TN+FN\right)\right)}}$
Error Rate (ER)	The sum of all inaccurate predictions, based on the number of observations.	$ErR=\frac{\left(FP+FN\right)}{\left(TP+FN+TN+FP\right)}$
Jaccard Index (JI)	True positives, whether they were genuine or projected, were outweighed by false positives.	$Jac=\frac{TP}{\left(TP+FP+FN\right)}$
Classification Success Index (CSI)	By averaging the symmetric class measure for each individual class	$CSI=\frac{TP}{\left(TP+FP\right)}+\frac{TP}{\left(TP+FN\right)}\;–100$
g-Mean (g-M)	Aggregation of Both Sensitivity and Specificity Measures of the Classifiers	$g-Mean=\sqrt{\text{Sensitivity}*\text{Specificity}}$
Kappa (Ka)	It indicates how well the machine learning classifier matched ground truth data.	$Kappa=(\frac{TP+TN}{100}-\;Eacc$)/(1-Eacc) Eacc=(((TP+FP)/100)*(TP+FN)/100+(((TN+FP)/100)*((TN+FN)/100))

The reported results are presented in the succeeding tables.

Table 6 shows the reported results of the classifiers based on performance metrics such as Accuracy, F1, MCC, ER, JI, CS I, g-M, and Ka for HT DRT without FSEL techniques. It is visible from Table 6 that the BLDC reported the best Acc of 82.32 %, an F1 of 88.32 %, with a low ER of 17.67 %. The BLDC also demonstrates a moderate JI value of 79.08 %, g-M of 69.23, MCC 0.5757 and Ka of 0.5326. The RFC reported low results with an Acc of 58.563 %, high ER of 41.437 % and an F1 of 69.87 % along with a JI of 53.703 %. The MCC and Ka of the RFC are 0.1459 and 0.1167, respectively. A lower g-M of 45.12 % places the RFC as a lower edge classifier.

**Table 6:** Reported results for HT DRT without FSEL.

Classifiers	Parameters							
	Acc	F1	MCC	ER	JI	CSI	g-M	Ka
BLDC	**82.32**	**88.32**	**0.5757**	**17.67**	**79.08**	**78.24**	**69.23**	**0.5326**
NBC	58.565	69.63	0.1651	41.43	53.41	45.99	45.94	0.1302
RFC	58.563	69.87	0.1459	41.437	53.703	45.87	45.12	0.1167
DTC	79.005	86.89	0.3495	20.99	76.83	74	61.087	0.3443
SVM(Linear)	75.138	83.271	0.4135	24.862	71.337	68.784	60.359	0.3729
SVM(Poly)	64.641	74.19	0.3173	35.36	58.974	55.21	53.17	0.2521
SVM(RBF)	67.403	76.49	0.3631	32.597	61.93	59.049	55.579	0.2942

Table 7 shows the reported results of the classifiers based on DFA based DRT without FSEL. It is identified from Table 7 that the NBC attained high accuracy of 87.29 %, along with F1 of 91.98 % with a low ER of 12.71 %. The NBC displays a good value of the JI of 85.16 %, g-M of 75.45 %, MCC of 0.6312 and Ka of 0.6162. The BLDC reported inferior results with an Acc of 55.24 %, a high ER of 44.76 %, an F1 of 66.93 %, and a JI of 50.31 %. The MCC and Ka of the BLDC are 0.09604 and 0.0747, respectively. A lower g-M of 42.51 % pushes the BLDC to the bottom of the classifier results.

**Table 7:** Reported resultsfor DFA DRT without FSEL.

Classifiers	Parameters							
	Acc	F1	MCC	ER	JI	CSI	g-M	Ka
BLDC	55.24	66.93	0.09604	44.76	50.31	40.98	42.51	0.0747
NBC	**87.29**	**91.982**	**0.6312**	**12.71**	**85.16**	**84.35**	**75.45**	**0.6162**
RFC	67.95	77.69	0.2955	32.05	63.52	59.15	53.33	0.2533
DTC	60.77	70.53	0.2811	39.23	54.48	50.07	50.93	0.2126
SVM(Linear)	85.08	91.02	0.4677	14.92	83.53	82.06	71.71	0.4678
SVM(Poly)	61.32	73.076	0.1153	38.68	57.57	49.69	44.11	0.0988
SVM(RBF)	60.22	70.24	0.2568	39.78	54.14	49.05	49.92	0.1956

Table 8 shows the reported results of classifiers for LSLR based DRT without FSEL. From Table 8 that SVM (Linear) Classifier is attained high accuracy of 79.01 %, with F1 of 86.89 %, and with ER of 20.99 %. The SVM (Linear) classifier demonstrates a good value of JI of 76.82 %, g-M of 61.08 %, low MCC of 0.3495 and Ka of 0.3443. The DTC is with low Acc of 56.35 %, and high ER of 43.65 % and F1 of 68.52 % along with JI of 52.12 %. The MCC and Ka of DTC is 0.0678 and 0.0549 respectively. The Lower CSI of 42.48 % moves the DTC into the bottom level of the classifier results.

**Table 8:** Reported results for LSLR DRT without FSEL.

Classifiers	Parameters							
	Acc	F1	MCC	ER	JI	CSI	g-M	Ka
BLDC	73.48	81.53	0.4457	26.52	68.83	67.03	60.53	0.3821
NBC	58.56	68.08	0.2808	41.44	51.61	47.45	50.48	0.2031
RFC	56.35	68.27	0.0871	43.65	51.82	42.52	42.18	0.0696
DTC	56.35	68.52	0.0678	43.65	52.12	42.48	41.26	0.0549
SVM(Linear)	**79.01**	**86.89**	**0.3495**	**20.99**	**76.82**	**74**	**61.08**	**0.3443**
SVM(Poly)	63.53	74.81	0.1564	36.47	59.75	52.83	46.43	0.1357
SVM(RBF)	76.79	84.67	0.4179	23.21	73.41	70.88	61.44	0.3866

Table 9 depicts the classifiers' performance analysis based on the HT DRT with EHO FSEL. As presented in Table 9, the NBC reported top Acc of 81.76 %, with an F1 of 88.58 % and with an ER of 18.24 %. The NBC achieves the MCC and Ka values of 0.448 and 0.4369. The SVM (Linear) Classifier reported an inferior Acc of 56.35 %, a high ER of 43.65 % and an F1 of 66.94 %, along with the JI of 50.31 %. The MCC and Ka of the SVM(Linear) classifier are 0.1831 and 0.1364, respectively. The Lower g-M value of 46.36 % positioned the SVM(Linear) Classifier into the lowermost edge of the classifiers.

**Table 9:** Reported results for HT DRT with EHO FSEL.

Classifiers	Parameters							
	Acc	F1	MCC	ER	JI	CSI	g-M	Ka
BLDC	69.06	78.62	0.3074	30.94	64.77	60.63	54.14	0.2666
NBC	**81.76**	**88.58**	**0.4448**	**18.24**	**79.5**	**77.42**	**66.21**	**0.4369**
RFC	69.06	77.95	0.381	30.94	63.87	61.19	56.69	0.3139
DTC	81.76	88	0.5522	18.24	78.57	77.46	68.31	0.5134
SVM(Linear)	56.35	66.94	0.1831	43.64	50.31	43.22	46.36	0.1364
SVM(Poly)	57.45	68.04	0.1931	42.56	51.57	44.77	46.93	0.1461
SVM(RBF)	59.66	69.19	0.2905	40.34	52.9	48.91	51.05	0.2133

Table 10 exhibits the classifier's performance for DFA based DRT with EHO FSEL. As presented in Table 10, the SVM(Linear) Classifier is positioned at a top Acc of 92.26 %, with an F1 of 95.36 % and a low ER of 7.74 %. The MCC and ka of 0.7209 and 0.7203 are obtained for SVM (Linear) Classifier. The NBC is placed at a low Acc of 60.22 %, a high ER of 39.78 % and an F1 of 69.49 %, along with the low MCC and Ka of 0.3151 and 0.2296, respectively. The Lower CSI of 50.01 % pushes the NBC to the lowest level.

**Table 10:** Reported results for DFA DRT with EHO FSEL.

Classifiers	Parameters							
	Acc	F1	MCC	ER	JI	CSI	g-M	Ka
BLDC	75.13	83.87	0.3271	24.87	72.22	68.69	57.56	0.3096
NBC	60.22	69.49	0.3151	39.78	53.24	50.01	52.05	0.2296
RFC	86.18	91.52	0.5449	13.82	84.37	83.12	73.69	0.5426
DTC	91.71	94.88	0.7378	8.29	90.25	89.86	83.11	0.7321
SVM(Linear)	**92.26**	**95.36**	**0.7209**	**7.74**	**91.139**	**90.73**	**86.68**	**0.7203**
SVM(Poly)	67.4	77.73	0.2344	32.6	63.58	58.23	50.77	0.2069
SVM(RBF)	85.08	90.18	0.6474	14.92	82.11	81.86	72.89	0.6018

Table 11 Reported results for the LSLR-based DRT with EHO FSEL. As tabulated in Table 11, the SVM(RBF) Classifier reported a top Acc of 90.61 % and a low ER of 9.39 %. The MCC and ka of 0.7228 and 0.7099 are ascertained by the SVM(RBF) Classifier. The SVM(poly) Classifier is placed with a low Acc of 62.43 % and a high ER of 37.57 %, along with the low MCC and Ka of 0.2964 and 0.2291, respectively. The lower g-M of 51.86 % is the reason for the classifier's lower ebb performance.

**Table 11:** Reported results for LSLR DRT with EHO FSEL.

Classifiers	Parameters							
	Acc	F1	MCC	ER	JI	CSI	g-M	Ka
BLDC	62.43	71.91	0.3155	37.57	56.12	52.56	52.56	0.2403
NBC	65.74	75.2	0.3281	34.26	60.25	56.66	53.86	0.2641
RFC	87.84	92.56	0.5943	12.16	86.16	85.16	76.79	0.5926
DTC	85.63	91.15	0.5332	14.37	83.75	82.38	72.67	0.531
SVM(Linear)	88.95	93.19	0.6422	11.05	87.26	86.47	78.55	0.6385
SVM(Poly)	62.43	72.13	0.2964	37.57	56.41	52.28	51.86	0.2291
SVM(RBF)	90.61	94.11	0.7228	9.39	88.88	88.51	80.76	0.7099

Table 12 displays the reported results of classifier for the HT-based DRT with CS FSEL. As shown in Table 12 that the DTC is placed at top Acc of 83.97 % and with a low ER of 16.03 %. The DTC ascertains the MCC and ka of 0.5574 and 0.5364. The NBC is positioned with a low Acc of 65.74 % and a high ER of 34.26 %, along with the low MCC and Ka of 0.2168 and 0.1881, respectively. The Lower g-M value of 49.58 % is the reason for the classifiers poor results.

**Table 12:** Reported results for HT DRT with CS FSEL.

Classifiers	Parameters							
	Acc	F1	MCC	ER	JI	CSI	g-M	Ka
BLDC	80.66	87.81	0.4261	19.34	78.26	75.97	64.65	0.4159
NBC	65.74	76.33	0.2168	34.26	61.72	55.95	49.58	0.1881
RFC	67.95	77.34	0.3321	32.05	63.05	59.39	54.66	0.2779
DTC	**83.97**	**89.75**	**0.5574**	**16.03**	**81.41**	**80.15**	**70.52**	**0.5364**
SVM(Linear)	73.48	82.35	0.3408	26.52	70	66.46	57.16	0.3122
SVM(Poly)	67.95	77.86	0.2772	32.05	63.75	59.07	52.64	0.2404
SVM(RBF)	70.71	79.53	0.3795	29.29	66.02	63.16	57.28	0.3234

Table 13 displays the classifier's performance for DFA based DRT with CS FSEL. As mentioned in Table 13 that the SVM(RBF) Classifier is reported at top Acc of 91.71 % and with a low ER of 8.29 %. The MCC and ka of 0.7378 and 0.7321 are ascertained by the SVM(RBF) Classifier. The BLDC is again positioned at low Acc of 69.06 % and a high ER of 30.94 %, along with the low MCC and Ka of 0.2343 and 0.2124 respectively. The Lower g-M of 51.29 % is the reason for the classifier's poor results.

**Table 13:** Reported results for DFA DRT with CS FSEL.

Classifiers	Parameters							
	Acc	F1	MCC	ER	JI	CSI	g-M	Ka
BLDC	69.06	79.25	0.2343	30.94	65.64	60.5	51.29	0.2124
NBC	70.71	79.53	0.3795	29.29	66.02	63.16	57.28	0.3234
RFC	69.06	79.1	0.2527	30.94	65.43	60.49	52.04	0.2267
DTC	90.05	94.03	0.6409	9.95	88.75	88.08	82.24	0.6404
SVM(Linear)	85.63	91.15	0.5332	14.36	83.75	82.38	72.67	0.531
SVM(Poly)	75.69	84.39	0.3171	24.31	73.006	69.48	57.54	0.3039
SVM(RBF)	**91.71**	**94.88**	**0.7378**	**8.29**	**90.25**	**89.86**	**83.11**	**0.7321**

Table 14 explores the reported results of classifier for the LSLR based DRT with CS FSEL. As presented in Table 14, the SVM(RBF) Classifier reported high Acc of 91.71 % and with a low ER of 8.29 %. The MCC and ka of 0.7378 and 0.73206 are ascertained by the SVM(RBF) Classifier. The BLDC is again reported at low Acc of 56.35 % and a high ER of 43.65 %, along with the low MCC and Ka of 0.1445 and 0.1109, respectively. The Lower CSI of 42.83 % is the reason for the classifier's poor performance.

**Table 14:** Reported results for LSLR DRT with CS FSEL.

Classifiers	Parameters							
	Acc	F1	MCC	ER	JI	CSI	g-M	Ka
BLDC	56.35	67.48	0.1445	43.65	50.93	42.83	44.76	0.1109
NBC	71.82	80.89	0.3385	28.18	67.92	64.31	56.33	0.3021
RFC	65.74	76.15	0.2354	34.26	61.49	56	50.355	0.2018
DTC	58.56	69.87	0.1459	41.44	53.71	45.88	45.12	0.1167
SVM(Linear)	58.56	69.38	0.18414	41.44	53.125	46.14	46.74	0.1432
SVM(Poly)	60.77	71.02	0.2428	39.23	55.06	49.57	49.48	0.1889
SVM(RBF)	**91.71**	**94.88**	**0.7378**	**8.29**	**90.25**	**89.86**	**83.11**	**0.73206**

**Fig. 10:**
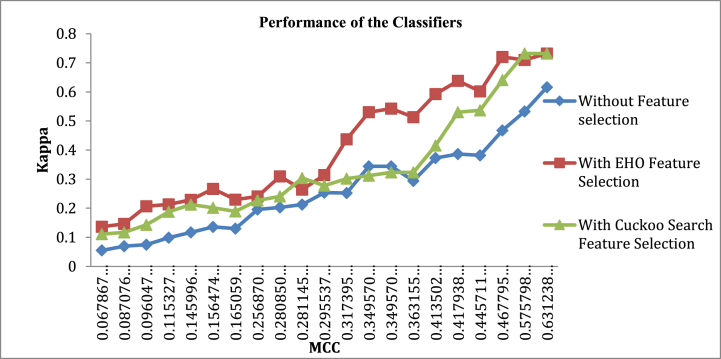
Reported results with and without FSEL for MCC and Ka Parameters.

Fig. 10 shows the reported results with and without FSEL for MCC and Ka parameters. As examined in Fig. 10 that all three methods plot almost linear in the initial values of MCC from 0 to 0.3. In the case of EHO, the FSEL method follows steep slope values of Kappa for MCC values from 0.3 to 0.73 when compared to the plot without FSEL methods. The CS FSEL method explores the middle path of the two methods in the incremental Kappa and MCC. The average MCC and Ka for FSEL methods are 0.299 and 0.26375. In the CS FSEL method, the average MCC and Ka are 0.36454 and 0.3361, respectively. The higher average values of MCC and Ka are displayed by the EHO FSEL method, and the values are 0.4434 and 0.4083, correspondingly.

To further investigate the MCC and Kappa characteristics shown in Fig. 10 of the manuscript, we considered the graph as three regions: region one with MCC ∈ [0, 0.3], region two with MCC ∈ [0.3, 0.45], and region three with MCC ∈ [0.45, 0.73]. The average slope value is the highest (1.85) in region two with the EHO FSEL. Likewise, the slope value is lowest (0.45) in region one without FSEL. Similarly, the maximum attained slope of 1.07 for CS FSEL is at region 2. These values reveal the effectiveness of EHO and CS FSEL algorithms. This is also evident in the higher accuracy rates of 92.26 % and 91.71 % achieved by EHO and CS FSEL algorithms, respectively.

**Fig. 11:**
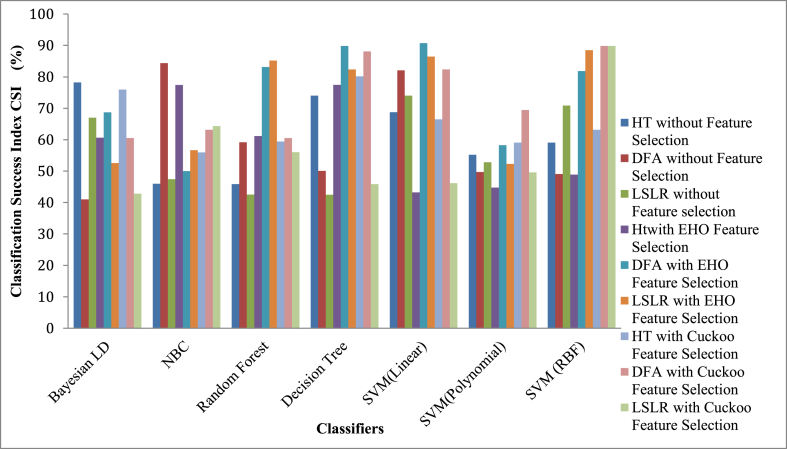
Reported results with and without FSEL for Classification Success Index (CSI) Parameter.

Fig. 11 shows the reported results of Classifiers with and without FSEL for the Classification Success Index (CSI) Parameter. As exhibited by Fig. 11, the DFA DRT with EHO FSEL for SVM (Linear) Classifier attains a higher value of CS Index 90.73 %.

The low CS index of 40.98 % is reached by the BLDC for the DFA DRT without any FSEL Techniques. The average Classification Success Index (CSI) across the classifier among three DRT without FSEL reached 59.035 %. The average CSI value of 68.581 % is attained for EHO FSEL techniques. As in the CS FSEL method the average CSI value for the classifier is slotted to 65.15 %.

### 5.1 Computational complexity

Next, we analyse classifiers based on their computational complexity (CompCo), which is determined by the input size O(n). A CompCo of O(1) indicates less complexity. When the CompCo increases log(n) times for every increase in ‘n', it is represented as O(logn). Thus, increasing the number of inputs leads to higher CompCo. Additionally, all the classifiers in this paper are hybrid, as they classify the DimRed data using FSEL techniques.

Table 15 depicts the CompCo for all the classifiers between various DRT with and without FSEL method. It is witnessed from Table 15 that the NBC and SVM (Linear) classifier are possess comparatively less CompCo across the three DRT without FSEL methods. SVM (Linear) and NBC reported reasonable complexity for the EHO and CS FSEL techniques across the employed DRT. The LSLR DRT with EHO FSEL involves high CompCo during classification. The RFC across the DRT with and without FSEL techniques involves high CompCo but the achieved Acc of the classifier is at the lower edge. SVM Linear and RBF reported good results with moderate CompCo.

**Table 15:** Computational complexity of the classifiers.

Classifiers	Without FSEL			With EHO FSEL			With CS FSEL		
	DRT			DRT			DRT		
	HT	DFA	LSLR	HT	DFA	LSLR	HT	DFA	LSLR
BLDC	O (n^3^)	O(n^3^)	O(n^4^)	O(n^6^)	O(n^6^)	O(n^7^)	O(n^3^ log n)	O(n^3^ log n)	O(n^4^ log n)
NBC	O(n^2^)	O(n^2^)	O(n^3^)	O(n^5^)	O(n^5^)	O(n^6^)	O(n^2^ log n)	O(n^2^ log n)	O(n^3^ log n)
RFC	O(n^2^ log n)	O(n^2^ log n)	O(n^3^ log n)	O(n^5^ log n)	O(n^5^ log n)	O(n^6^ log n)	O(n^3^ log 2n)	O(n^3^ log 2n)	O(n^4^ log 2n)
DTC	O(nlog n)	O(nlog n)	O(n^2^ log n)	O(n^4^ log n)	O(n^4^ log n)	O(n^5^ log n)	O(n^2^ log 2n)	O(n^2^ log 2n)	O(n^3^ log 2n)
SVM(Linear)	O(n^2^)	O(n^2^)	O(n^3^)	O(n^5^)	O(n^5^)	O(n^6^)	O(n^2^ log n)	O(n^2^ log n)	O(n^3^ log n)
SVM(Poly)	O(n^3^)	O(n^3^)	O(n^4^)	O(n^6^)	O(n^6^)	O(n^7^)	O(n^3^ log n)	O(n^3^ log n)	O(n^4^ log n)
SVM(RBF)	O(nlog n)	O(nlog n)	O(n^2^ log n)	O(n^4^ log n)	O(n^4^ log n)	O(n^5^ log n)	O(n^2^ log 2n)	O(n^2^ log 2n)	O(n^3^ log 2n)

Table 16 shows the comparison of our research work with the some of the bench mark results on the lung cancer classification from MGED using binary classifiers. The research performed by Lin Ke et al. [47] used a swarm optimization that works with filter wrapper and gene selection for population initialization, and also a ranking criterion was employed to transform the population initialization of Genetic Algorithm (GA) and Ant Colony Optimization (ACO), respectively. The Decision Tree C4.5 classifier is thus embedded with the highly complex metaheuristic feature extraction and selection method, which has high computational complexity and obtained a slight increase of 0.96 % in accuracy. Our method has comparatively lesser computational complexity than Lin Ke's work but achieved an appreciable accuracy of 92.26 %. Our research is also progressing continuously, and we are working on improving the FSEL and feature extraction steps with the application of Wavelet transforms, STFT, scalogram and CNN classifiers. So there is a scope for obtaining a higher accuracy, and our future research is in that direction only.

**Table 16:** Comparison with previous work.

SI.No	Author (with year)	Database	Classifier	Classes	Acc in (%)
1	Gavin (2002) [48]	LH2 Dataset	Gene expression ratios	Adeno, Meso	90
2	Hasseb (2015) [49]	Nat Lib of Medicine and Kent Ridge	SVM, MLP, RBFN	Adeno, Meso	91 91 89
3	Hanan et al. (2021) [50]	LH2 Dataset	DTC with feature fusion	Adeno, Meso	85
4	Peng et al.(2009) [51]	Affymetrix Human Gene Atlas U95Av2 microarray	SVM (RBF) with gene based feature selection	Adeno, Meso	94
5	Surbhi et al. (2022) [52]	TCGA	Deep learning and CNN	Adeno, Meso	92
6	Minca et al. (2007) [53]	Dataset MIT www.genome.wi.mit.edu\MPR\lung	SVM, Naive Bayes, KNN, DTC	Adeno, Meso	94 90 75 91
7	Lin (2022) [47]	LH2 Dataset	DT C 4.5	Adeno, Meso	93
8	Daniel et al. (2020) [54]	LH2 Dataset	Error Minimum Classifier	Adeno, Meso	90
9	Federica (2021) [55]	TCGA and GEO	Cox Regression	Adeno, Meso	90
10	Our Research	LH2 Dataset	DFA DR with EHO based FSEL and SVM (Linear) Classification	Adeno, Meso	92

Using gene expression datasets in machine learning and deep learning classification tasks is powerful, but several limitations exist. The gene expression datasets are large and contain more genes compared to the number of samples. This problem called the “Curse of Dimensionality,” can lead to challenges in model training and interpretation. It can increase the risk of overfitting, require more computational resources, and make it harder to identify the most informative genes. Gene expression data can be noisy due to various sources of variation like experimental noise, technical biases, and biological variability. Noisy data can negatively impact the performance of machine learning models, leading to decreased accuracy and reliability. Gene expression datasets are often generated from multiple batches or experimental runs, which can introduce systematic variations unrelated to the biological signal. These batch effects can confound the classification task, making distinguishing between true biological differences and technical artifacts difficult. Obtaining gene expression data from biological experiments can be costly and time-consuming. Gene expression datasets capture the complex and heterogeneous nature of biological systems leading to reduced accuracy and difficulties in generalizing to new datasets. Also, careful FSEL methods must be incorporated to select relevant genes for classification can be challenging. Addressing these limitations requires careful preprocessing strategies for ensuring robust and meaningful analysis in machine learning and deep learning classification methodologies.

In this research, we have employed bio-inspired FSEL methods for the performance enhancement of various classifiers like BLDC, NBC, RFC, DT and SVM. Thus the research aids in the early detection of lung cancer from MGED. The machine learning model utilised in the research can be applied to other MGED of prostate, ovarian, colon, CNS, and Leukemia. The analysis of MGED can be applied to person-specific treatment and therapy. Also, the classifier model is self-sustained, evident from the MCC and Kappa performance characteristic plots.

## 6 Conclusion

Among the cancer ailments, lung cancer poses a major threat to human beings nowadays. The early detection of lung cancer extends the patient's life expectancy. Therefore, the aforementioned problem is regarded as the prime objective of this paper. This paper utilizes microarray gene expression incorporated with machine learning techniques to attain high accuracy with a minimum error rate. The most suitable outcomes are obtained for DFA DRT cascaded with the EHO FSEL method. For the above combination, the SVM(Linear) classifier attained the top accuracy of 92.26 %. The future direction of this research may explore other bio-inspired FSEL methods to enhance the classification accuracy. Also, convolutional neural networks and deep learning neural networks may be employed to extend this research further.

## Data availability

The datasets used and analysed during the current study are available from the corresponding author on reasonable request.

## CRediT authorship contribution statement

**Karthika M S:** Conceptualization, Data curation, Formal analysis, Investigation, Methodology, Project administration, Resources, Software, Validation, Visualization, Writing – original draft, Writing – review & editing. **Harikumar Rajaguru:** Conceptualization, Data curation, Formal analysis, Investigation, Methodology, Project administration, Resources, Software, Supervision, Validation, Writing – review & editing. **Ajin R. Nair:** Conceptualization, Data curation, Formal analysis, Investigation, Methodology, Resources, Software, Validation, Visualization, Writing – review & editing.

## Declaration of competing interest

The authors declare the following financial interests/personal relationships which may be considered as potential competing interests:Harikumar Rajaguru reports a relationship with Bannari Amman Institute of Technology that includes: employment.
